# diAcCA, a Pro-Drug for Carnosic Acid That Activates the Nrf2 Transcriptional Pathway, Shows Efficacy in the 5xFAD Transgenic Mouse Model of Alzheimer’s Disease

**DOI:** 10.3390/antiox14030293

**Published:** 2025-02-28

**Authors:** Piu Banerjee, Yubo Wang, Lauren N. Carnevale, Parth Patel, Charlene K Raspur, Nancy Tran, Xu Zhang, Ravi Natarajan, Amanda J. Roberts, Phil S. Baran, Stuart A. Lipton

**Affiliations:** 1 Neurodegeneration New Medicines Center, Department of Molecular & Cellular Biology, The Scripps Research Institute, La Jolla, CA 92037, USA; pbanerjee@scripps.edu (P.B.); yubowang@scripps.edu (Y.W.); lcarnevale@scripps.edu (L.N.C.); papatel@scripps.edu (P.P.); craspur@scripps.edu (C.K.R.); nantran@scripps.edu (N.T.); xuzhang@scripps.edu (X.Z.); 2 Socrates Biosciences, Inc., Camarillo, CA 93012, USA; ravi.natarajan@socratesbio.com; 3 Behavioral Core, The Scripps Research Institute, La Jolla, CA 92037, USA; aroberts@scripps.edu; 4 Department of Chemistry, The Scripps Research Institute, La Jolla, CA 92037, USA; pbaran@scripps.edu; 5 Department of Neurosciences, School of Medicine, University of California, San Diego, CA 92093, USA

**Keywords:** Alzheimer’s disease, Nrf2 transcriptional pathway, carnosic acid, therapeutic drug for neurodegeneration

## Abstract

The antioxidant/anti-inflammatory compound carnosic acid (CA) is a phenolic diterpene found in the herbs rosemary and sage. Upon activation, CA manifests electrophilic properties to stimulate the Nrf2 transcriptional pathway via reaction with Keap1. However, purified CA is readily oxidized and thus highly unstable. To develop CA as an Alzheimer’s disease (AD) therapeutic, we synthesized pro-drug derivatives, among which the di-acetylated form (diAcCA) showed excellent drug-like properties. diAcCA converted to CA in the stomach prior to absorption into the bloodstream, and exhibited improved stability and bioavailability as well as comparable pharmacokinetics (PK) and efficacy to CA. To test the efficacy of diAcCA in AD transgenic mice, 5xFAD mice (or littermate controls) received the drug for 3 months, followed by behavioral and immunohistochemical studies. Notably, in addition to amyloid plaques and tau tangles, a hallmark of human AD is synapse loss, a major correlate to cognitive decline. The 5xFAD animals receiving diAcCA displayed synaptic rescue on immunohistochemical analysis accompanied by improved learning and memory in the water maze test. Treatment with diAcCA reduced astrocytic and microglial inflammation, amyloid plaque formation, and phospho-tau neuritic aggregates. In toxicity studies, diAcCA was as safe or safer than CA, which is listed by the FDA as “generally regarded as safe”, indicating diAcCA is suitable for human clinical trials in AD.

## 1. Introduction

Alzheimer’s disease (AD) is the sixth leading cause of death in the United States [1]. It is also the most common cause of dementia among older adults. It is a relentless and progressive neurodegenerative disorder that predominantly affects the hippocampus and cortex. Its primary hallmark is the accumulation of extracellular plaques of amyloid-β (Aβ) and intracellular aggregates of phosphorylated-tau (pTau). Cognitive decline occurs due to neuronal synaptic damage, which partially occurs due to excessive oxidative and nitrosative stress, in part triggered by oligomeric Aβ peptide [2,3,4,5,6,7,8,9].

All cells maintain an equilibrium between oxidation and reduction (termed redox balance), the disturbance of which can contribute to various disorders [10,11,12]. Electrophiles can regulate this redox state of cells by initiating cellular defense systems against oxidative stress and inflammatory reactions. Electrophiles, however, can also cause damage and even lead to the development of cancer. In this regard, carnosic acid (CA) is a compound that is not electrophilic on its own, but becomes an electrophile in tissues undergoing oxidative/inflammatory insult, a mechanism designated as a “pro-electrophilic drug” or PED [13,14,15]. CA is a phenolic diterpene which is naturally found in the herbs rosemary (Rosmarinus officinalis) and common sage (Salvia officinalis) [16,17,18,19]. We showed that the primary action of CA was activation of the Nrf2 (nuclear factor erythroid 2-related factor 2) anti-inflammatory/antioxidant transcriptional pathway, while also stimulating HSF-1 (heat shock factor 1) to stimulate chaperone expression [20,21,22,23,24,25]. CA has been previously shown to activate a large number of Nrf2-induced phase II antioxidant and anti-inflammatory enzymes and other gene products. Critically, RNA interference (RNAi) of Nrf2 completely abrogated the neuroprotective effect of CA, implicating this pathway as the primary cytoprotective mechanism [26].

Multiple studies have shed light on the relation between Nrf2 and neurodegenerative disorders such as AD, including evidence that Nrf2 is downregulated in human AD brain [27,28,29]. Along these lines, our group reported the neuroprotective effect of CA treatment in transgenic mouse models of AD [30]. In that study, transnasally administered CA increased synaptic markers, decreased inflammatory markers, and limited both amyloid plaques and phospho-tau aggregates, in addition to improving learning and memory on behavioral tests [30]. Nonetheless, a major limitation with pure CA (and drugs of similar structure with *ortho*- or *para*-phenol groups) as a potential therapeutic is that it is highly unstable and easily oxidized, yielding a very short shelf-life and rendering it unsuitable for oral administration. In an effort to overcome these limitations, we synthesized multiple stable derivatives of CA, amongst which a di-acetylated derivative (diAcCA) showed the greatest potential as a drug candidate.

Seeking to test an oral compound of sufficient stability and bioavailability, we show here that diAcCA is completely converted to CA in the stomach and then absorbed into the bloodstream as CA. diAcCA exhibits excellent bioavailability, ~20% improved over CA itself. When converted to CA, the drug displays excellent pharmacokinetic (PK) properties in the blood, reaching neurotherapeutic levels in the brain in less than 1 h. Moreover, no toxicity was noted to the digestive system after oral administration of diAcCA. To prove the efficacy of diAcCA in a manner that could be adapted to a human clinical trial, in the present study we treated the 5xFAD transgenic mouse model of AD (vs. wild-type (WT) littermates) with oral diAcCA vs. placebo (vehicle) control. WT and 5xFAD mice received the drug or vehicle by oral gavage (designated here as PO) for 3 months, following which behavioral studies, immunohistochemical analysis, and transcriptomic assessment were performed. We report here that diAcCA rescues neuronal and synaptic loss, decreases Aβ plaques and pTau aggregates, and reduces gliosis and neuroinflammation in the hippocampus of treated animals compared to controls. Furthermore, NanoString transcriptomic data showed that diAcCA treatment ameliorates inflammatory pathways. Behavioral assessments showed that diAcCA treatment improves spatial learning and memory on the Morris water maze test and conditioned fear test. Taken together, these results indicate that this bioavailable derivative of CA has therapeutic potential for the treatment of human AD. Moreover, since CA, which is the product of diAcCA metabolism, is on the Food and Drug (FDA) generally regarded as safe (GRAS) list, human clinical trials with diAcCA should be greatly expedited.

## 2. Materials and Methods

### 2.1. 5xFAD Transgenic Mice

All animal experiments were performed according to the guidelines of IUCAC at The Scripps Research Institute (Scripps Research), La Jolla, California, USA [Protocol number: 17-0022-3, date of approval: 2023/11/01-2026/11/01].

The 5xFAD mice bear five mutations linked to AD. Three of these are in the App695 gene [Swedish (APP K670N/M671L), Florida (I716V), London (V717I)], and two are in the PSEN1 gene [L286V, M146L]. Male 5xFAD breeder mice were obtained from Jackson Laboratories (#034848-JAX, Bar Harbor, ME, USA) and crossed with female 6XC57BL6 mice. Genotyping was performed by PCR. First-generation pups were used for all experiments with 5xFAD transgenic (Tg) and WT littermate control mice.

### 2.2. diAcCA Treatment

diAcCA or control vehicle was administered three times per week for 3 months to 5xFAD Tg or WT littermate control mice starting at approximately 5 months of age by oral gavage (designated here as “per os” or PO). Three different doses were used (10 mg/kg, 20 mg/kg, or 50 mg/kg). diAcCA was dissolved in olive oil vehicle, a non-polar organic solvent. For vehicle control, we administered olive oil alone. A total of 45 animals were used in this study, with 7–8 animals in each group.

### 2.3. Pharmacokinetic Analysis

We tested both CA and diAcCA compounds by oral gavage/PO at 10 mg/kg. Analysis was performed by LC-MS in the Scripps Florida Drug Development Core (Jupiter, FL, USA). The compounds were formulated in olive oil. CA went into the oil with minimal stirring, but diAcCA required longer stirring. Blood was collected in hematocrit tubes and centrifuged. Oily plasma was noticeable at 30 min, maximal at 1–2 h, and started to decrease at about 6 h. Oil absorbs from the gut and is emulsified in the blood but does not absorb through the portal vein as most drugs do. It is absorbed as chylomicrons through the lymphatic system. Drug is therefore likely absorbed via enterocyte passage and through chylomicron absorbance, complicating the early time points of the PK curves.

For the IV dose, we tried several formulations. Aqueous or micelle-based formulations were still not in solution after 8 h of stirring. Therefore, we decided to pre-weigh the CA into a vial and, in the vivarium, rapidly dissolve the compound in DMSO and then add Tween80/saline. The compound was dosed within five minutes of being dissolved, and the final concentrations were 5% DMSO and 95% of a 5% Tween80 solution in saline. The IV results obtained in this manner yielded a more traditional curve shape and had a half-life of ~12 h, in general agreement with the past literature [26,31,32,33,34]. Note that the clearance for the IV is lower than the oral clearance. Oral clearance calculations assume 100% bioavailability. When you correct the oral clearance for bioavailability (CL*%F), the values are almost identical.

### 2.4. Immunohistochemistry

For euthanasia, animals were deeply anesthetized with isoflurane and then transcardially perfused with ice-cold phosphate-buffered saline (PBS) followed by 4% paraformaldehyde (PFA) for fixation. Brains were isolated and post-fixed in 4% PFA overnight at 4 °C. The next day, brains were washed and transferred to 30% sucrose at 4 °C until sectioning. Every brain then received a masked code for the following steps, and the code was only broken after all quantifications were completed.

Sagittal sections (40 μm-thick) were prepared using a vibratome (Leica VT1200S) and incubated in blocking buffer (5% goat serum, 0.5% Triton X-100 in PBS) for 1 h at room temperature to prevent non-specific binding. Following blocking, floating sections were incubated in their respective primary antibody solutions on a shaker overnight at 4 °C. Antibodies against the following proteins were used in this study: NeuN (D4G4O, 1:500, rabbit mAb #24307, Cell Signaling Technology, Danvers, MA, USA), Synapsin I (D12G5, 1:500, rabbit mAb #5297, Cell Signaling Technology), phosphor (Ser202, Thr205)-tau (AT8, 1:500, mAb #MN1020, ThermoFisher Scientific, Waltham, MA, USA), glial fibrillary acidic protein (GFAP) (GA5, 1:500, MAB360, Millipore Sigma, Carlsbad, CA, USA), ionized calcium binding adaptor molecule 1 (Iba1) (EPR16589, 1:1000, #ab178847, Abcam, Waltham, MA, USA), human nuclear antigen (HuNu) (1:500, mouse mAb #191181, Abcam), and caspase-1 (1:50, rabbit pAb #AB1871, Sigma Aldrich, St. Louis, MO, USA). For Aβ staining, floating sections were pre-treated with 90% formic acid (28905, Thermo Scientific) for 1 min at room temperature. They were then rinsed and blocked, following which they were incubated in anti-β-amyloid 17–24 antibody (4G8, 1:1000, #800708, BioLegend, San Diego, CA, USA) on a shaker overnight at 4 °C.

**Figure 1:**
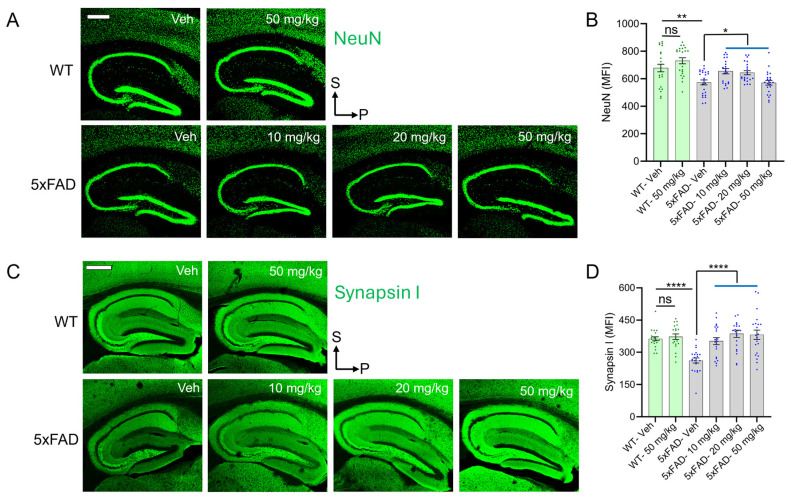
diAcCA treatment rescues deficits in neuronal and synaptic density in 5xFAD mice. Quantitative immunohistochemistry (Q-IHC) of hippocampal sections was performed on 5xFAD and WT littermate mice treated with diAcCA (10 mg/kg, 20 mg/kg, or 50 mg/kg) or olive oil vehicle (Veh). (**A**) Representative images showing NeuN antibody staining of neurons in WT and 5xFAD mice hippocampal sections. (**B**) Bar graph quantifying NeuN mean fluorescence intensity (MFI). diAcCA restored NeuN MFI (representing neuronal number) to near normal levels in 5xFAD mice. (**C**) Representative images showing Synapsin I antibody staining in WT and 5xFAD treatment groups. (**D**) Bar graph quantifying Synapsin I MFI. diAcCA treatment restored Synapsin I MFI (located in the presynaptic terminal of the synapse) to near normal levels in 5xFAD mice. Each dot on the graph represents the value of brain sections from single mouse and error bars are SEM (n = 4–7 mice/group; 3–6 sections/brain). Scale bar, 500 μm. Significance tested by Student’s *t*-test of grouped data (* *p* < 0.05, ** *p* < 0.005, **** *p* < 0.0001; ns, not significant).

**Figure 2:**
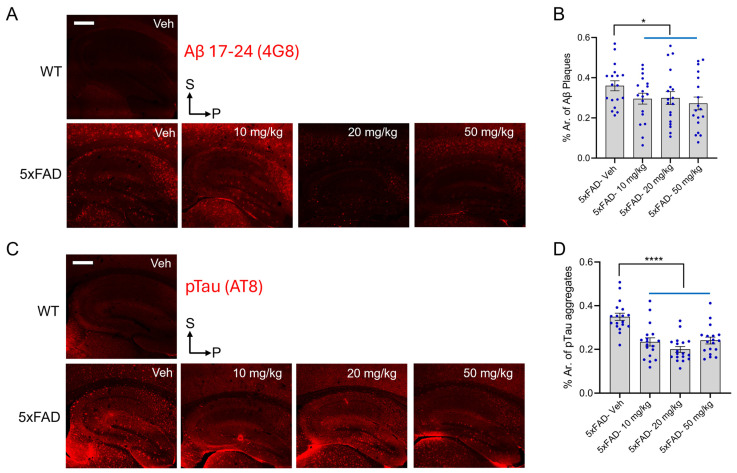
diAcCA treatment ameliorates AD-related aggregated proteins in 5xFAD mice. Q-IHC was performed on hippocampal sections from WT and 5xFAD littermate mice treated with vehicle (Veh) or diAcCA (10 mg/kg, 20 mg/kg, 50 mg/kg). (**A**) Representative images showing Aβ(17–24) antibody (clone 4G8) staining of Aβ plaque-like aggregates in WT and 5xFAD treatment groups. (**B**) Bar graph quantifying percent area (% Ar.) of Aβ aggregates in 5xFAD control and treatment groups. diAcCA treatment significantly reduced the area of Aβ aggregates in 5xFAD mice. (**C**) Representative images showing pTau antibody (AT8) staining in WT and 5xFAD treatment groups. (**D**) Bar graph quantifying pTau aggregates in 5xFAD control and treatment groups. diAcCA treatment dramatically reduced the area of pTau aggregates in 5xFAD mice. Each point on the graphs represents the value from brain sections from a single mouse and error bars are SEM (n = 4–7 mice/group; 3 sections/brain). Scale bar, 500 μm. Student’s *t*-test of grouped data (* *p* < 0.05, **** *p* < 0.0001).

**Figure 3:**
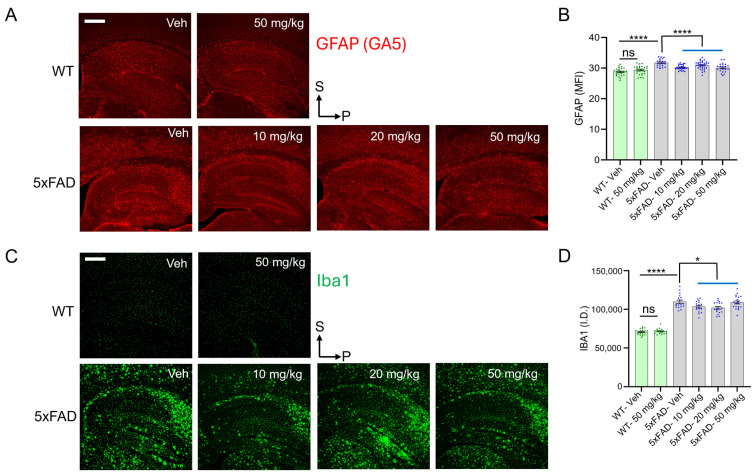
diAcCA treatment improves neuroinflammation in 5xFAD mice. Q-IHC was performed on hippocampal sections from WT and 5xFAD littermate mice treated with vehicle (Veh) or diAcCA (10 mg/kg, 20 mg/kg, 50 mg/kg). (**A**) Representative images showing GFAP antibody (GA5) staining of astrocytes from the WT and 5xFAD treatment groups. (**B**) Bar graph quantifying GFAP MFI. diAcCA treatment reduced GFAP MFI, representing astrocytic cells in 5xFAD mice. (**C**) Representative images showing Iba1 antibody staining microglial cells in WT and 5xFAD treatment groups. (**D**) Bar graph quantifying Iba1 ID. diAcCA treatment improved IBA ID, representing microglial density in 5xFAD mice. Each point on the graphs represents the value from brain sections from a single mouse and error bars are SEM (n = 4–7 mice/group; 3–4 sections/brain). Scale bar, 500 μm. Student’s *t*-test of grouped data (* *p* < 0.05, **** *p* < 0.0001; ns, not significant).

The next day, immunostaining was performed on brain sections after 3 washes in PBS and incubation in the appropriate secondary antibody solution on a shaker for 2 h at room temperature. Secondary antibodies included Alexa 488 (A11008, 1:500, goat anti-rabbit, Life Technologies, Carlsbad, CA, USA) and Alexa 555 (A21424, 1:500, goat anti-mouse, Life Technologies). The sections were again washed with PBS 3 times, mounted on Superfrost plus slides (12-550-15, Fisher Scientific, Waltham, MA, USA) and dried at room temperature. The slides were then coated with Vectashield plus antifade mounting medium, stained with nuclear dye DAPI (H-2000, Vector Laboratories, San Francisco, CA, USA), and a coverslip (12541057, Fisher Scientific) was applied. For each brain, 3–6 images were acquired for each individual marker using a confocal fluorescence microscope (Nikon Eclipse Ti2) (Figure 1, Figure 2 and Figure 3, Supplementary Figure S1). Post image processing, quantification was performed using Fiji ImageJ (1.54f) [35]. For the Iba1-stained slides, a mask was made of cells co-labeled for HuNu and Iba1, and then caspase 1 staining was measured. For quantifying caspase-1 in Iba1/ HuNu+ cells, an outlier test was used to exclude from the analysis a small set of images that did not pass this quality control measure. For quantification, NeuN, Synapsin I, and GFAP staining were compared between groups by mean fluorescence intensity (MFI), Iba1 and caspase-1 staining was compared by integrated density (ID), Aβ and pTau staining were compared by % area of plaques/aggregates, as previously described [30,36].

### 2.5. Morris Water Maze Behavioral Test

The Morris water maze test is used to evaluate spatial learning and memory skills in rodents [37]. In the experimental setup, mice are placed in a circular tank containing opaque water maintained at a consistent temperature of 21 ± 1 °C. The mice undergo 2 trials each day for a period of 4 days during which they are trained to find a hidden platform placed somewhere within the tank. The platform acts as an escape for the mice from swimming. The starting point of the mice in the tank is randomized; however, the location of the platform remains unchanged for a given mouse. After entering the water, the mice are given a maximum of 90 s to navigate to the platform and climb it. If a mouse is unable to find the platform in this duration, the experimenter offers gentle guidance to help it in reaching the platform. On day 5, after the training phase is over, a “probe trial” is conducted in which the platform is removed from the water maze. The mice are allowed to swim for a period of 60 s to observe if they learned to use spatial cues to navigate to safety (i.e., swim to the area in which the platform had been located and then spend more time in this quadrant of the pool than the other quadrants). After each trial, the mice are given a 10 min rest period in which they are transferred to a new cage with a warm towel. Data are analyzed using Noldus EthoVision software (version XT 17, Leesburg, VA, USA) with parameters measured for “latency to escape” for the acquisition trials and “quadrant time” as well as “distance and velocity of swimming” in the probe test.

### 2.6. Contextual and Cued Conditioned Fear Test

Contextual and cued learning processes in mice rely on the hippocampus and amygdala, respectively. Fear conditioning is used to evaluate both of these processes [38]. During the conditioning period, mice are trained to associate a context (distinctive environment) and a conditioned stimulus (CS, neutral stimulus consisting of a tone and light) with an unpleasant foot shock to which the mice learn to respond by freezing their movement [39]. Here, we evaluated hippocampal function using the contextual and cued conditioned fear test. For this, mice were exposed to the context or CS alone, without the shock, to see if they still exhibit freezing behavior, which is the conditioned behavior. If a mouse freezes during either the context or cued tests, it points to an established association between the specific stimulus (the environment or the tone/light) and the shock, and acts as an indication of hippocampal learning. Plexiglas Freeze Monitor Chambers (Size: 26 cm × 26 cm × 17 cm, Med Associates, Inc., St. Albans, VT, USA), equipped with lights on opposite walls, speakers, and shockable grid floors, were used to conduct the conditioning. The chamber was further enclosed with sound-proof boxes. Real-time digital videos were acquired during the sessions to detect freezing behavior.

On day 1, the mice were acclimatized to the chambers in a 5 min session with no shock. The next day, they were subjected to both context (to monitor contextual fear conditioning) and a CS comprising a 30 s, 80 dB, 3000 Hz sound accompanied by white light (to monitor cued fear conditioning). This stimulus was accompanied by a foot shock (scrambled current, 0.60 mA, 2 s). In this 3 min period, the mice were subjected to a single shock given in the last 2 s of exposure to the 30 s light/tone. On day 3, a 5 min trial took place in the original training chamber (context test) and contextual conditioning was evaluated by observing freezing behavior. On day 4, the mice were exposed to the CS to evaluate cued conditioning. For this experiment, the context was modified with a white Plexiglas floor and white curved wall insert, and mice were tested for 3 min with no cues, followed by an exposure to the CS (light + tone) for the next 3 min.

### 2.7. NanoString Analysis

The hippocampus/cortex were isolated from the brain and RNA isolation was performed immediately using the Quick-RNA Miniprep kit (76211-580, VWR, Radnor, PA, USA). RNA was then analyzed by the Salk Institute Single-Cell & Spatial Omics Core for the nCounter Neuropathology panel (NanoString, Bruker Spatial Biology, Inc., Seattle, WA, USA). The list of genes included in the NanoString analysis is provided as Supplementary Information (Table S3). The analysis was performed using the following packages on R studio software version 4.2.3: readr 2.1.5, dplyr 1.1.4, clusterProfiler 4.6.2, org.mm.eg.db 3.16.0, ggplot2 3.5.1, and stringr 1.5.1. Differentially expressed genes (DEGs) having log_2_ change > 0.5 and *p*-value < 0.05 were defined as upregulated or downregulated, depending on their direction of change.

### 2.8. Toxicology Analysis

Starting at 5 months of age, WT 6XC57BL6 mice were given either the highest dose of diAcCA used in this study (50 mg/kg) or olive oil vehicle three times a week for three months by oral gavage/PO. Following the treatment, the esophagus and stomach were isolated, and 5 μm-thick coronal and sagittal sections were cut from the esophagus. The stomach was opened along the greater curvature, cleaned with PBS, and pinned flat. Two 5 μm-thick sagittal sections were collected, one extending from the cardiac region to the duodenum, and the other from the forestomach to the fundic area. All sections were made using a rotary microtome (HM325-#387821, Fisher Scientific) and mounted on SuperfrostPlus slides (#1255015, Fisher brand). The sections were stained by hematoxylin and eosin stain (H&E) [Abcam H&E kit, ab245880], mounted with permount, dried overnight at room temperature, and then imaged. Acquired images were analyzed in a masked fashion for inflammation by a board-certified pathologist.

### 2.9. Transplantation into Humanized Mouse Brain

Human induced pluripotent stem cell (hiPSC)-derived microglia (hiMG) were transplanted into humanized mouse brains as we have previously described [36]. Briefly, NSG-SGM3 (NSGS) triple transgenic mice were purchased from Jackson Laboratories (013062) that have been shown to support the engraftment of human myeloid cells. hiMG were collected on the 24th day of differentiation and resuspended in PBS [40]. The hiMG were exposed to oligomeric αSyn (final concentration: 0.5 µg/2 µL injection volume), or to oligomeric αSyn + anti-αSyn Ab scFv (final concentration: 100 ng/2 µL injection volume) just prior to injection [41,42,43,44,45]. Stereotactically, the cells were injected into anesthetized, immobilized female mice (4-week old) since they show improved engraftment over males [46]. The injection into the ventricle was set to deliver 200,000 cells in 2 µL PBS over a 10 min period. The following coordinates from the bregma were used for the injection: dorsoventral (DV) 2.4 mm; anteroposterior (AP) −0.3 mm; mediolateral (ML) −1 mm. Two analgesic administrations were given to the mice, one immediately after transplantation, and another after 24 h. Two-weeks post transplantation, the brains were harvested. Isolated brains were placed into 4% PFA overnight for fixation and then transferred to 30% sucrose in PBS prior to freezing. Cryostat sections (20 µm in thickness) were subsequently cut for analysis.

### 2.10. Statistics

GraphPad Prism 10 and R studio version 4.2.3 (GraphPad Prism Software, San Diego, CA, USA) were used for all statistical tests and data visualization plots. A *p* value less than 0.05 was considered statistically significant. Values presented are mean, and error bars represent standard error of the mean (SEM) or standard deviation (SD). Sample size for all data and statistical tests used are listed in the figure legends.

## 3. Results

### 3.1. diAcCA Exhibits Improved Bioavailability and Pharmacokinetics Compared to CA

Extensive pharmacokinetic (PK) and bioavailability data for simultaneous plasma and brain samples are already available for CA after administration via both the PO and intravenous (IV) routes, with brain levels reaching approximately 10% of the plasma level at peak values [26,31,32,33,34]. In the present study, to compare CA and diAcCA, we assessed plasma levels of each and found that diAcCA was completely converted to CA in the stomach prior to uptake into the bloodstream; therefore, plasma levels could be used to predict the brain levels from the prior extensive plasma/brain PK data. We also determined the stability and bioavailability of diAcCA compared to CA. For this purpose, individual mice were administered CA or diAcCA IV or via oral gavage/PO. For CA, the doses were 3 mg/kg for the IV route and 10 mg/kg for oral gavage/PO, and for diAcCA 10 mg/kg by oral gavage/PO. Plasma concentrations were evaluated at ten different time points over the next 72 h (raw data shown in Tables S1 and S2, Supplementary Materials). As expected of a pro-drug destined to become CA upon exposure to stomach acid, after administration of diAcCA, only CA and not the parent compound diAcCA was detected in the blood. This finding supported the premise that diAcCA was entirely metabolized in the stomach to CA and, consequently, then absorbed into the bloodstream as CA.

After oral gavage, PK analysis revealed peak plasma levels of CA within ~15 min of administration of diAcCA and a long half-life (t_1/2_) of over 12 h (Table 1, Supplementary Figure S2A–C, and Supplementary Tables S1 and S2); moreover, CA derived from diAcCA administration exhibited a mean C_max_ of 5.75 µM, nearly 30% greater than the mean C_max_ of 4.45 µM after oral gavage of CA itself. Additionally, as calculated from area under the curve (AUC) values, diAcCA showed 20% better bioavailability than CA (PO) (Table 1). Importantly, we also observed that CA was highly unstable over time, with a shelf life of only a couple of months even if kept in the dark and cold. In contrast, diAcCA has been stable in our hands for over two years. Thus, diAcCA represents an improved drug candidate over CA from the point-of-view of stability and bioavailability, with excellent PK comparable to CA, and, because diAcCA is metabolized to CA in the stomach, with similar absorption, distribution, metabolism, and excretion (ADME) to that of CA [31].

### 3.2. diAcCA Toxicology

Extensive toxicity and safety data are already published on CA [47,48]. Based on the fact that diAcCA is totally metabolized to CA in the stomach prior to entering the bloodstream and the prior finding that CA has one of the highest therapeutic indices of any currently available drug (LD_50_ = 7.1 g/kg) [48], in a preliminary meeting with the US Food and Drug Administration (FDA), we were asked to limit our toxicity studies of diAcCA to the esophagus and stomach. Accordingly, in light of our PK findings, we selected a dose of 50 mg/kg to assess safety of the drug after oral administration to WT mice. We performed a non-GLP toxicology analysis of vehicle (olive oil) or drug administered PO three times a week for 3 months starting at 5 months of age. Following this period, sections from the stomach and esophagus were stained with H&E and examined blindly by a board-certified toxicologic pathologist. The results revealed that diAcCA actually improved baseline inflammation in the esophagus of WT animals, and the inflammation in the stomach was similar to that of mice that received vehicle (Supplementary Figure S2D,E). Thus, diAcCA is as safe or safer than CA in vivo.

### 3.3. diAcCA Treatment Rescues Neuronal Synaptic Loss in 5xFAD Mice

The extensive existing PK data obtained on plasma and brain samples for CA, correlated with prior in vivo efficacy data for CA [26,33,49], allowed us to calculate the therapeutic dose range that we wanted to test here for diAcCA. Based on our prior studies, we knew that ~10–20 µM CA in the plasma produces ~1–2 µM CA in the brain, which is neuroprotective [26]. Therefore, in the present study, we bracketed this estimated efficacious dose to provide a dose–response of diAcCA action on neuronal damage and synaptic loss as well as behavioral effects. For this purpose, we administered either vehicle (olive oil) or drug PO three times a week for 3 months at doses of 10 mg/kg, 20 mg/kg, or 50 mg/kg to WT and 5xFAD littermate mice starting at ~5 months of age, producing a predicted C_max_ in the brain of ~0.6, ~1.2, and ~3 µM, respectively. We chose to initiate treatment at 5 months of age because 5xFAD mice begin to exhibit synaptic loss and behavioral abnormalities similar to those observed in human AD at early stages [50]. Following drug administration, neurobehavioral tests were performed (see below), following which, animals were sacrificed, the brains removed, and hippocampal/cortical sections prepared for quantitative confocal immunohistochemistry (Q-IHC) using antibodies against NeuN (a marker of neuronal nuclei) and Synapsin I (a presynaptic marker).

WT hippocampal sections displayed strong NeuN and Synapsin I signals, but these immunofluorescent signals were significantly reduced in sections from 5xFAD vehicle-treated mice (Figure 1). Quantifying the mean fluorescence intensity (MFI) of NeuN and Synapsin I staining showed that diAcCA significantly improved both neuronal density (Figure 1A,B) and presynaptic density (Figure 1C,D) in 5xFAD mice receiving the drug. Moreover, since 5xFAD mice of this age manifest neuronal and synaptic loss [50], these results show that diAcCA rescues these phenotypes in this AD transgenic mouse model.

### 3.4. diAcCA Treatment Reduces Aβ Accumulation and pTau Aggregates in 5xFAD Mouse Brain

Aβ accumulates in plaques throughout the human brain in AD, starting in the neocortex, expanding to allocortex (including the hippocampus), then into subcortical nuclei, including the striatum, finally involving the brainstem and cerebellum. Similarly, multiple brain regions of 5xFAD mice, including the hippocampus, accumulate Aβ peptide [50,51,52]. Thus, we assessed the effect of diAcCA on Aβ levels in the hippocampus of WT and 5xFAD mice that received vehicle or varying doses of the drug (10 mg/kg, 20 mg/kg, 50 mg/kg). Q-IHC was performed with Aβ17–24 antibody (4G8), and the percent area of Aβ accumulation was compared between 5xFAD vehicle and drug-treated mice. Our data showed that diAcCA treatment significantly reduced the area of Aβ accumulation in the hippocampus of animals that received the drug (Figure 2A,B).

As in human AD brain, 5xFAD mice are also known to manifest high levels of pTau in their brains, beginning at a later stage than Aβ accumulation [53]. Hence, we also compared the percentage area of pTau aggregates in 5xFAD vehicle- and drug-treated mice by Q-IHC with pTau antibody (AT8). We observed a remarkable decrease in the area of pTau aggregates in the hippocampus of mice at each treatment dose (Figure 2C). Taken together, these data show that diAcCA decreases the hallmark aggregated proteins in the hippocampus of 5xFAD mice.

### 3.5. diAcCA Treatment Decreases Astrocytosis and Microglial Neuroinflammatory Markers in 5xFAD Mouse Brain

Astrocytosis and neuroinflammation play key roles in the pathophysiology of AD and are believed to exacerbate the progression of the disease [54,55]. Microglial and astrocytic activation have been observed in various regions of 5xFAD mouse brain, including the hippocampus [56,57]. We therefore performed Q-IHC using antibodies against GFAP (GA5, a glial marker) and Iba1 (a microglial marker). WT hippocampal sections showed strong GFAP and Iba1 signals, which were significantly increased in sections from 5xFAD vehicle-treated mouse brain (Figure 3). Quantifying the MFI of GFAP and ID of Iba1 staining revealed that diAcCA significantly improved both astrocytic (Figure 3A,B) and microglial density (Figure 3C,D) in 5xFAD mice receiving the drug. Collectively, these data suggest that diAcCA reduces astrocytic and microglial inflammation in this AD transgenic mouse model.

### 3.6. diAcCA Treatment Inhibits Inflammatory Pathways on mRNA Analysis

**Figure 4:**
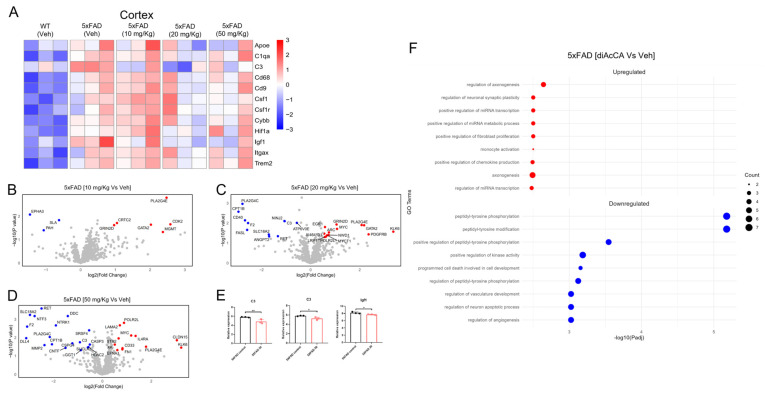
nCounter^®^ NanoString analysis of 5xFAD mouse brain after diAcCA treatment. (**A**) Heatmap showing gene expression of selected DAM-related genes in each group. (**B**–**D**) Volcano plots showing DEGs for each treatment group compared to vehicle (Veh) control. Grey dots represent genes not significantly affected, while red dots represent upregulated genes, and blue dots, downregulated genes. (**E**) Relative expression of DAM-related genes C3 and Igf1E, which were significantly lower in 5xFAD-treatment groups compared to 5xFAD-Veh. Values are mean ± SD, significance tested by Student’s *t*-test (* *p* < 0.05, ** *p* < 0.005). (**F**) GO terms associated with DEGs for 5xFAD-diAcCA treated compared with 5xFAD-Veh. Circle size corresponds to number of genes associated with each GO term (n = 3 for each group).

To elucidate the molecular underpinnings of the effect of diAcCA on neuroinflammation, we next analyzed disease-associated microglia (DAM) signatures in WT and 5xFAD mouse brains using nCounter^®^ biomarker analysis (NanoString, Bruker Spatial Biology, Inc., Amsterdam, The Netherlands). RNA was isolated from the hippocampus/cortex of WT and 5xFAD mice that had received three-month vehicle or diAcCA treatment, and a NanoString neuropathology gene expression panel was performed (Figure 4). Heatmap analysis revealed expression of DAM-related genes in 5xFAD vs. WT mouse brain; however, we also observed a dose-dependent decrease in DAM signature with diAcCA treatment in 5xFAD brains (Figure 4A).

Considering all transcripts detected by NanoString nCounter analysis, Volcano plots highlight the differentially expressed genes (DEGs) that were up- or downregulated in treated 5xFAD mouse brain compared to vehicle-treated (Figure 4B–D). Amongst DAM genes, C3 and Igf1 were significantly downregulated in diAcCA-treated 5xFAD brain compared to vehicle-treated, indicating improvement in inflammatory gene expression in the higher treatment groups (20 or 50 mg/kg) (Figure 4E). Next, we performed gene ontology (GO) biological pathway analysis for the upregulated and downregulated genes in diAcCA-treated 5xFAD brain vs. vehicle-treated. We found that treatment resulted in upregulation of genes enriched in pathways associated with neuroprotection, such as regulation of axonogenesis, neuronal synaptic plasticity, as well as metabolic pathways, micro(mi)RNA transcription, and innate immunity. Conversely, downregulated genes were significantly enriched in pathways related to neuronal apoptotic processes, phosphorylation, and angiogenesis (Figure 4F). Taken together, these results support the notion that diAcCA improves inflammatory and neuroprotective pathways in the 5xFAD mouse brain.

### 3.7. CA Alleviates Protein Aggregate-Induced Inflammation in Human iPSC-Derived Microglia

**Figure 5:**
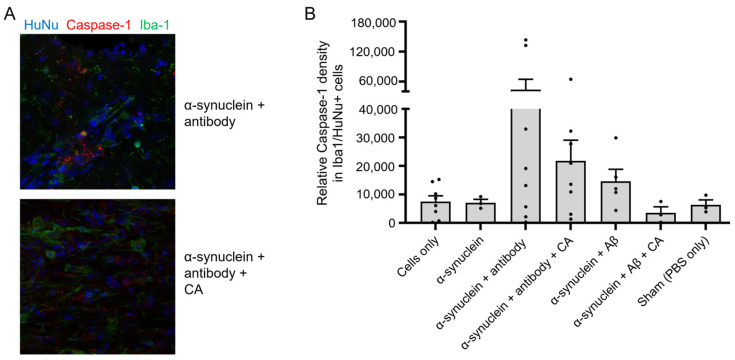
CA prevents protein aggregate/antibody-induced inflammatory response in hiMG. (**A**) Representative immunohistochemistry sections of hiMG transplanted into the brains of humanized mice after activation by α-synuclein aggregates plus cognate monoclonal antibody, as in Ref. [36]. CA (2 µM) prevented this effect. Anti-human nuclear antibody (HuNu, blue) was used to identify transplanted hiMG, which were also stained by Iba-1 (green); cleaved caspase-1 staining (red) signified NLRP3 inflammasome activation. (**B**) Bar graph quantifying immunohistochemical data. Each point represents the value from a section of brain and error bars are SEM (n = 3–8 sections from 17 mice). Log_10_ transformation normalized the distribution of datapoints, and a Student’s *t*-test revealed a significant decrease in cleaved/activated caspase-1 integrated density (ID) in response to α-synuclein/Aβ aggregates in the presence of CA (*p* = 0.0307).

Next, we investigated the underlying molecular basis for diAcCA, after being metabolized to CA, in ameliorating the neuroinflammatory response in the brain. For this purpose, we studied the effect of CA on hiPSC-derived microglia (hiMG). Notably, in addition to Aβ and pTau aggregates, over half of human AD brains also manifest α-synuclein aggregates, as found also in Lewy body dementia [58,59,60]. Along these lines, we had previously demonstrated that exposure of hiMG to α-synuclein and Aβ aggregates activated multiple inflammatory pathways, including the NLRP3 inflammasome, with consequent caspase-1 activation [36]. Moreover, exposure to monoclonal antibodies against either α-synuclein or Aβ increased the level of inflammation, possibly accounting at least in part for the development of amyloid-related imaging abnormality -edema and -hemorrhage (ARIA-E and ARIA-H), as observed in human clinical trials with similar antibodies [36]. Here, we transplanted hiMG previously activated by α-synuclein and Aβ aggregates in the presence or absence of their cognate antibodies into humanized mouse brain, and then assayed the brains for inflammatory markers 2 weeks later, as previously described [36]. We found that α-synuclein plus Aβ aggregates increased Iba1 and caspase-1 expression in transplanted hiMG, and that antibodies further increased this inflammatory response. Pretreatment of the hiMG with CA, however, significantly prevented this inflammatory reaction (Figure 5A,B). These findings support the notion that CA ameliorates the neuroinflammatory response of hiMG under conditions simulating exposure to AD-related protein aggregates or these aggregates in the presence of their cognate antibodies.

### 3.8. diAcCA Treatment Reverses Neurobehavioral Deficits 5xFAD Mice

**Figure 6:**
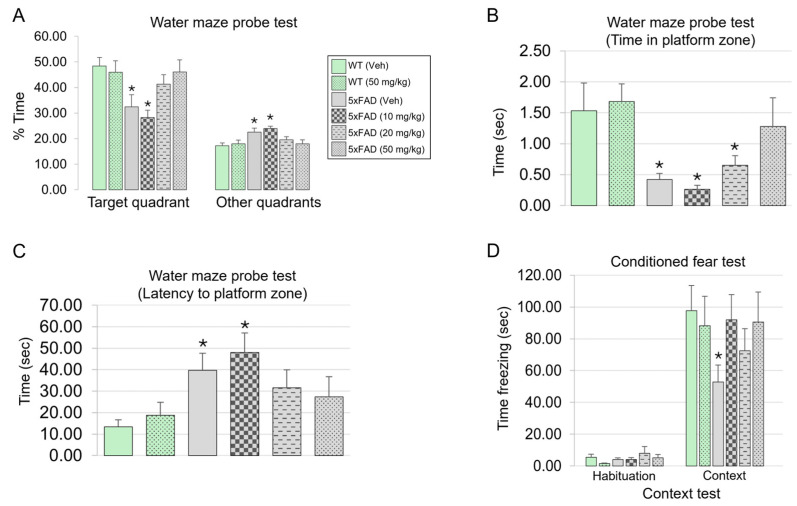
diAcCA treatment improves learning and memory in 5xFAD mice. (**A**–**C**) WT and 5xFAD littermate mice were assessed for spatial learning and memory in the Morris water maze probe test after treatment with diAcCA. (**A**) The 5xFAD vehicle-treated (Veh) and 10 mg/kg diAcCA-treated mice spent significantly less time in the target quadrant and more time in other quadrants compared to WT controls. However, the 5xFAD 20 mg/kg and 50 mg/kg-treated group mice spent a similar percentage of time in the target and other quadrants as the WT controls, signifying dose-dependent improvement. (**B**) The 5xFAD vehicle-treated, 10 mg/kg, and 20 mg/kg diAcCA-treated mice all spent significantly less time in the platform quadrant zone compared to WT control mice. In contrast, 5xFAD mice treated with 50 mg/kg diAcCA spent similar amount of time in the platform quadrant as WT control mice. (**C**) The 5xFAD vehicle-treated and 10 mg/kg diAcCA-treated mice took significantly more time to first enter the platform zone compared to WT controls, whereas 5xFAD20 mg/kg and 50 mg/kg-treated groups took a similar amount of time compared to WT controls. (**D**) On the context test of conditioned fear, 5xFAD-Veh mice, but not mice treated with diAcCA, froze for significantly less time than WT controls. Values are mean + SEM, n = 4–7 mice/group; * *p* < 0.05 vs. vehicle-treated WT by Fisher’s PLSD test. Note that no statistically-significant difference between WT and 5xFAD mice was noted on cued fear conditioning (Supplementary Figure S3).

Spatial learning and memory deficits are associated with human AD pathology, and, beginning at 5 months of age, 5xFAD mice also exhibit cognitive impairments reminiscent of human AD in this regard [50,51,52]. To delineate the effect of diAcCA on spatial memory, we performed the Morris water maze probe test, a widely used assessment for evaluating hippocampal-dependent memory [37]. After diAcCA or vehicle treatment of WT and 5xFAD mice for 3 months starting at 5 months of age, the animals were initially trained for 5 days to ensure learning of the spatial memory task, i.e., swimming to the location of a hidden platform in the opaque water maze that the mice could stand on. Subsequently, their memory for the task was measured using the “probe test”, monitoring how well each mouse recalled the location of the platform once it had been removed from the swimming tank. This was assessed by the amount of time spent in the proper quadrant of the tank that previously housed the platform (the “target” quadrant) vs. other quadrants. Figure 6A–C shows that 5xFAD mice receiving vehicle or the lowest dose of the drug (10 mg/kg) spent significantly less percentage time and total time in the target quadrant and took significantly longer to reach the target platform zone when compared to WT mice. In contrast, 5xFAD animals performed significantly better with regard to these criteria after treatment with the higher doses of diAcCA (≥20 mg/kg, or, for total time spent in the target quadrant, 50 mg/kg).

Another cognitive domain compromised in both human AD and in 5xFAD mice is represented by associative learning and memory, associated with hippocampal and amygdalar circuits. Here, we evaluated the context test of conditioned fear [38,39]. In this behavioral test, the mouse is placed in the environment in which it had learned to associate a neutral stimulus (tone) with an aversive stimulus (shock), resulting in freezing behavior if the animal remembers the fear association. In this context test of fear conditioning (Figure 6D), we observed that 5xFAD mice receiving only vehicle treatment displayed significantly less freezing behavior than WT mice. This deficit in the context test is consistent with hippocampal dysfunction in the 5xFAD mouse brain. Notably, this deficit was improved in the 5xFAD animals that received diAcCA treatment, since they showed freezing behavior similar to WT mice (Figure 6D). Taken together, these results demonstrate that learning and memory behavior is improved in 5xFAD mice after receiving diAcCA.

## 4. Discussion

PEDs represent therapeutic agents that are not electrophilic on their own, but become electrophiles in response to oxidative stress and inflammation. Critically, PEDs provide anti-inflammatory responses and neuroprotection by activating the Keap1/Nrf2 transcriptional pathway and subsequently increasing the levels of anti-inflammatory and antioxidant phase 2 enzymes [13,14]. Conventional electrophiles that are not pro-drugs like PEDs will react indiscriminately with thiol groups, for example, on glutathione (GSH) in normal cells, thereby depleting it and paradoxically lowering the threshold for toxicity in unstressed cells; electrophilic drugs also have the potential to activate cancerous cells. Because PEDs are only active in redox-stressed cells, they remain innocuous in normal tissue compared to electrophiles [13,15]. We have previously shown that CA crosses the blood–brain barrier, functions as a PED, and activates the Nrf2 transcriptional pathway [26,49]. However, CA is highly unstable with a short shelf life, and thus does not represent a viable drug for clinical and commercial purposes. Moreover, the therapeutic benefits of CA have been shown in vivo in multiple AD mouse models (hAPP-J20 and 3xTg AD mice) wherein CA rescued various AD-related histological findings in the hippocampus of treated mice and improved their neurobehavioral performance [30].

In the present study, our objective was to synthesize a pro-drug of CA (itself representing a pro-drug) in order to provide increased stability and better bioavailability, and thus a lead drug candidate for human therapy. In preliminary studies, we tested multiple congeners, amongst which the diacetyl derivative, diAcCa, displayed the most favorable properties in providing a stable shelf life (of over two years), increased oral bioavailability (by ~20%), and no toxicological issues. We show that orally-administered diAcCA is completely converted to CA in the digestive tract and absorbed into the blood as CA with no detectable diAcCA present. Note that while we dissolved diAcCA (or CA) in olive oil for oral gavage to animals, for future human use, a powder form is available that can be delivered PO in capsules. In our mouse studies, at similar doses, diAcCA displayed improved PK over CA itself, with an increased plasma C_max_ by ~30%. Based on our prior studies showing the relationship between plasma and brain levels of CA [26,30,33], we were able to calculate the bracketed doses of diAcCA to be tested for efficacy in AD model mice.

To test the efficacy of diAcCA in vivo, we used 5xFAD Tg mice and administered varying doses of the drug by oral gavage (PO) for three months, following which, immunohistochemical, transcriptomic, and behavioral studies were performed. Quantitative confocal immunohistochemistry on the hippocampus revealed that diAcCA treatment rescued neuronal and synaptic loss, decreased Aβ and pTau aggregates, and reduced astrocytosis and microglial inflammation. NanoString transcriptomic analysis revealed the molecular pathways whereby diAcCA treatment improved inflammation in the mouse brain. We further demonstrated that CA, as formed after diAcCA administration in vivo, alleviates pathological protein aggregate-induced inflammation in hiMG transplanted into humanized mouse brain. Finally, we found that diAcCA treatment improved behavioral performance on learning and memory tasks in 5xFAD mice.

## 5. Conclusions

Collectively, these findings underscore the promise of diAcCA as a potential therapeutic for human AD. Since before entering the bloodstream, diAcCA is completely converted to CA and CA is on the FDA’s GRAS list because of its longstanding safety record, human clinical trials testing diAcCA for AD should be expedited [61]. Moreover, diAcCA may prove useful not just for AD, but potentially for other neurodegenerative disorders and systemic maladies in which activation of the Nrf2 transcriptional pathway to alleviate inflammation and oxidative stress should modify disease pathogenesis [62,63,64,65,66].

## 6. Patents

The Scripps Research Institute has filed a patent for the composition and use of multiple CA derivatives in various neurodegenerative and systemic diseases. Phil S. Baran and Stuart A. Lipton are the named inventors on this patent.

## Figures and Tables

**Table 1:** Summary of PK data for CA and diAcCA *.

Drug, Route (Dose)	t_1/2_ (h)	t_max_ (h)	C_max_ (ng/mL)	C_max_ µM	AUC_last_ (μM.h)	AUC_INF_obs_ (min·ng/mL)	**AUC_%Extrap_**
CA, IV (3 mg/kg)	11.89	0.14	32,733	98.54	59.55	1,206,560	1.77
CA, PO (10 mg/kg)	16.32	1.50	1477	4.45	28.02	582,464	4.13
diAcCA, PO (10 mg/kg)	12.27	1.67	1910	5.75	34.46	698,697	1.91

* n = 3 mice/group.

## Data Availability

Additional data and codes used for analysis that support the findings are available from the corresponding author (S.A.L.) upon reasonable request.

## Supplementary Materials

The following supporting information can be downloaded at: https://www.mdpi.com/2076-3921/14/3/293/s1, Table S1: Pharmacokinetics of carnosic acid; Table S2: Pharmacokinetics of diAcCA; Table S3: List of genes in NanoString analysis; Figure S1: High-magnification images from hippocampus of vehicle-treated 5xFAD mice showing representative immunofluorescent staining for NeuN, Synapsin I, GFAP (GA5), Iba1, Aβ17–24 (4G8), and pTau (AT8) with and without DAPI; Figure S2: Pharmacokinetic and safety studies for diAcCA. Figure S3: Cued fear conditioning test.
