# Supplementary material for: diAcCA, a Pro-Drug for Carnosic Acid That Activates the Nrf2 Transcriptional Pathway, Shows Efficacy in the 5xFAD Transgenic Mouse Model of Alzheimer’s Disease
Source: Antioxidants (Basel). 2025 Feb 28;14(3):293. doi: 10.3390/antiox14030293 (PMC11939361; doi:10.3390/antiox14030293)
Supplement: Supplementary file 1 [file antioxidants-14-00293-s001.zip › Supplementary Files_Final/Supplementary Figures S1-S3 v6pb.pdf]

Supplemental Material for

diAcCA, a pro-drug for carnosic acid that activates the Nrf2 transcriptional pathway, shows efficacy in the 5xFAD Transgenic Mouse Model of Alzheimer's disease

Piu Banerjee, Yubo Wang, Lauren N. Carnevale, Parth Patel, Charlene K Raspur, Nancy Tran, Xu Zhang, Ravi Natarajan, Amanda Roberts, Phil S. Baran and Stuart A. Lipton

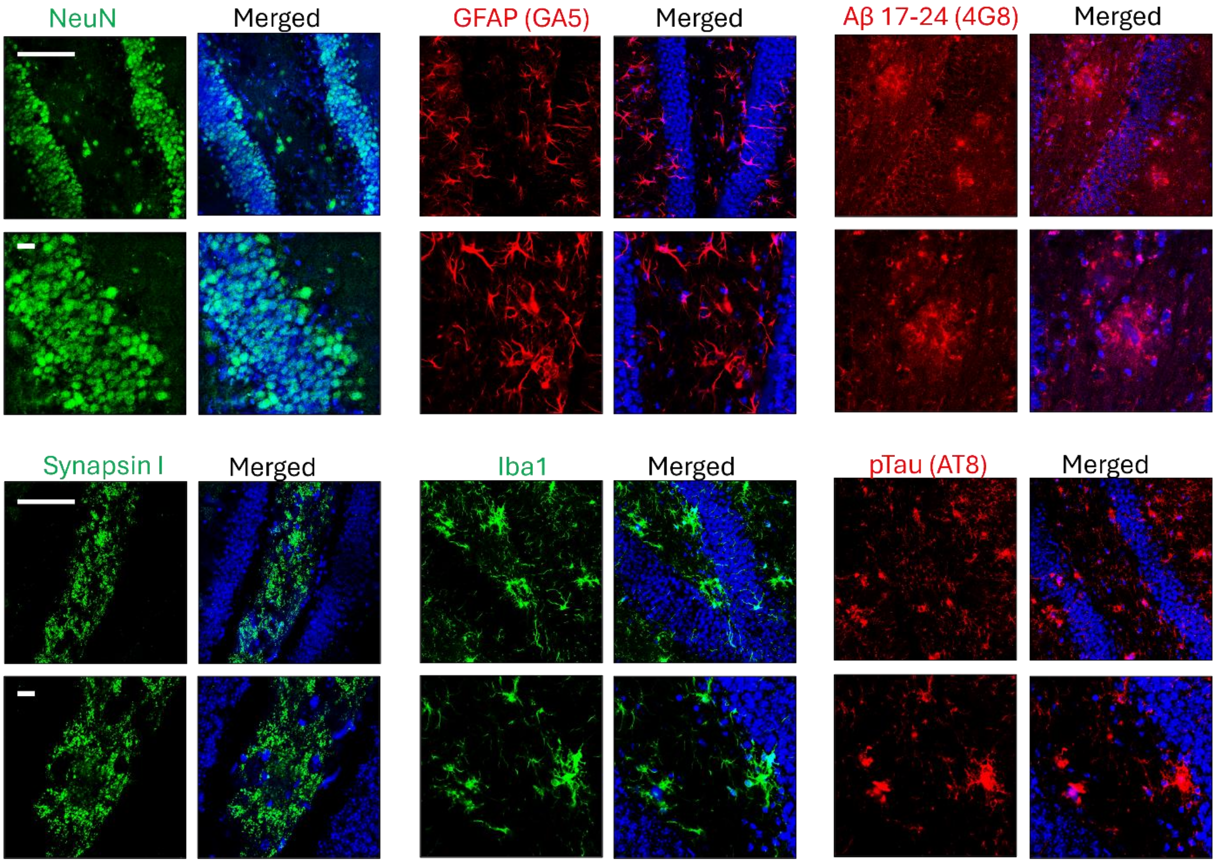

Supplementary Figure S1. High-magnification images from hippocampus of vehicle-treated 5xFAD mice showing representative immunofluorescent staining for NeuN, Synapsin I, GFAP (GA5), Iba1, Aβ 17-24 (4G8), pTau (AT8)] with (*right*) and without DAPI (blue, *left*). Scale bars, 100 µm.

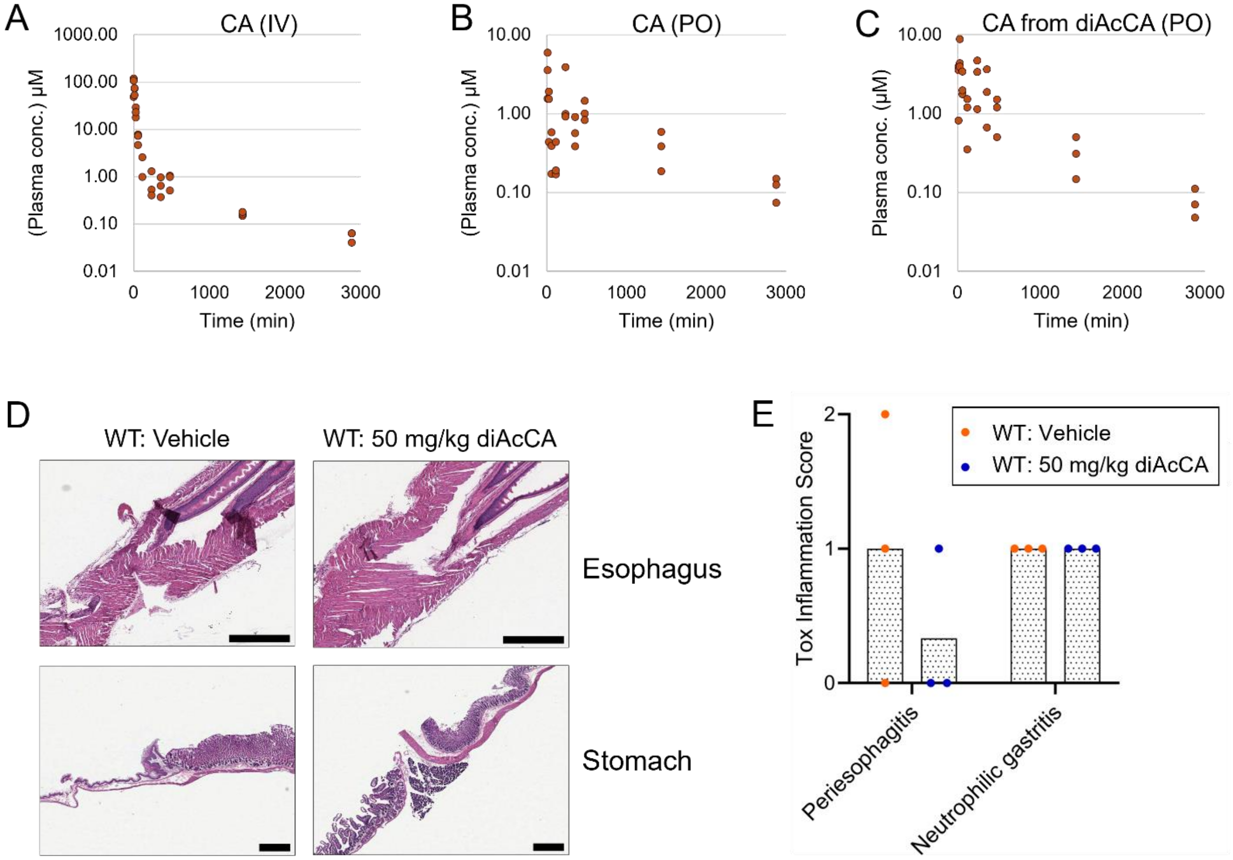

Supplementary Figure S2. Pharmacokinetic and safety studies for diAcCA. **(A, B)** Scatter plots display the concentration of CA in plasma of WT animals after intravenous (IV) administration of CA intravenously (5 mg/kg body weight) or oral gavage (PO, 10 mg/kg). After IV administration, the mean C_{max} was 98.5 µmoles/ml, while C_{max} was 4.45 µmoles/ml after PO administration. Average half-life for IV-CA and PO-CA were 11.89 hr and 16.32 hr, respectively. **(C)** Scatter plot shows the concentration of CA in plasma of WT animals that received PO diAcCA (10 mg/kg), with C_{max} of 5.75 µmoles/ml and an average half-life of 12.27 hr. **(D, E)** Non-GLP toxicology analysis on WT mice after treatment with vehicle (olive oil) or the highest concentration of diAcCA (50 mg/kg) used in these experiments. Representative esophagus and stomach lining sections (**D**) stained by hematoxylin & eosin (H&E) from two groups of mice. Scale: 500 µm. Bar graph (**E**) quantifying inflammation in esophagus (periesophagitis) and stomach (neutrophilic gastritis) by a standard tox inflammatory score ranging from 0 to 4 (0: inflammation within normal limits, 1: minimal inflammation, 2: mild inflammation, 3: moderate inflammation, 4: severe inflammation). diAcCA appeared to improve baseline inflammation in WT mice (n =3 animals in each group).

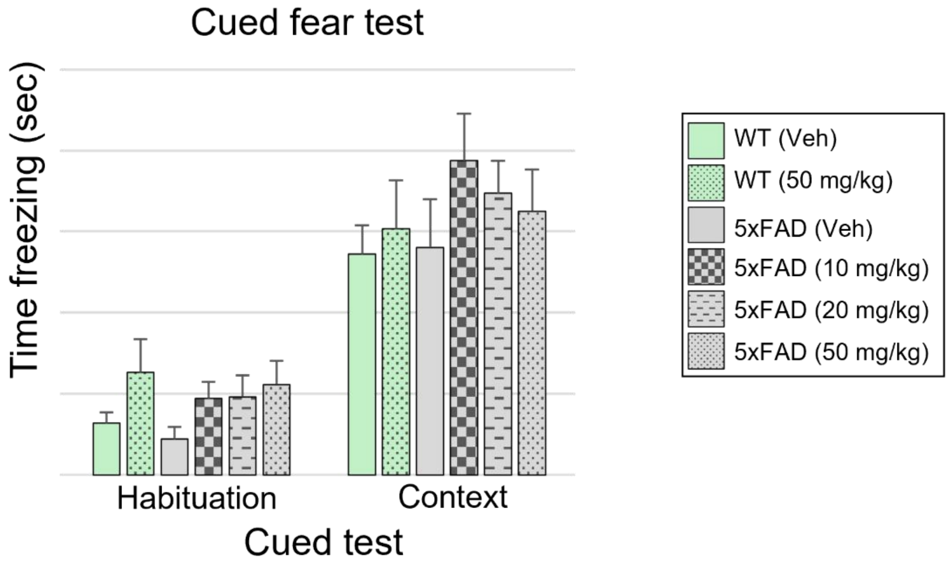

Supplementary Figure S3. Cued fear conditioning test. After the treatment course of diAcCA, WT and 5xFAD littermate mice were assessed on the cued fear conditioning test. No statistical significance difference between WT and 5xFAD mice was noted. Values are mean + SEM, n = 4–7 mice/group; Fisher's PLSD test.
